# Predictive value of hyperreflective foci for anti-VEGF therapeutic outcomes in different subtypes of diabetic macular edema: a retrospective analysis

**DOI:** 10.3389/fendo.2025.1648828

**Published:** 2025-10-16

**Authors:** Yalin Wang, Fengjiao Li, Miao Hao, Xianxian Kong, Weiyan Zhou

**Affiliations:** Department of Ophthalmology, Shandong Provincial Hospital Affiliated to Shandong First Medical University, Jinan, Shandong, China

**Keywords:** diabetic macular edema, diabetic retinopathy, hyperreflective foci, vascular endothelial growth factor, optical coherence tomography

## Abstract

**Objective:**

To analyze the correlation between optical coherence tomography (OCT) biomarkers and therapeutic outcomes in patients with different subtypes of diabetic macular edema (DME) following anti-VEGF treatment.

**Methods:**

A retrospective analysis was conducted on 113 patients diagnosed with DME and treated with intravitreal anti-VEGF therapy. Based on OCT characteristics, patients were categorized into three groups: cystoid macular edema (CME), diffuse retinal thickening (DRT), and serous retinal detachment (SRD). The primary outcome measures were the best-corrected visual acuity (BCVA), central macular thickness (CMT) and the number of hyperreflective foci (HRF) at each follow-up time point during the observation period. The secondary outcome measures included the status of other OCT biomarkers such as disorganization of retinal inner layers (DRIL), the inner segment/outer segment (IS/OS) disruption, and hard exudates at baseline. Additionally, the relationship between HRF, CMT and BCVA was analyzed.

**Results:**

The CME group had the worst BCVA (LogMAR) throughout (vs. DRT/SRD, all P<0.05), while DRT and SRD showed no significant difference (all P>0.05). For HRF, the DRT group had the fewest HRF across retinal layers from baseline to the end of follow-up (e.g., inner retina: 1.91 ± 1.22 at 12 months, ANOVA P<0.001; *post-hoc*: DRT vs. CME P<0.001, DRT vs. SRD P<0.001). The CME group exhibited the great reduction in HRF (inner retinal layer: -2.53 ± 2.14 vs. -1.07 ± 1.44 in DRT, P = 0.001; -2.36 ± 2.34 in SRD, P = 0.457) but recurrence at 6 months (351.78 ± 110.93 μm at 6 months vs. 330.03 ± 94.94 μm at 3 month). The SRD group maintained the highest HRF number, especially in outer retina (e.g., outer retinal layer: 1.97 ± 1.81 at 12 months vs. 0.66 ± 0.99 in DRT, P<0.001; 1.47 ± 0.97 in CME, P = 0.109). The DRT group had the lowest CMT at baseline (ANOVA P<0.001; *post-hoc*: DRT vs. CME P<0.001, DRT vs. SRD P<0.001). At 6 months, differences were mainly between DRT and CME (ANOVA P = 0.017; *post-hoc*: p=0.006). The SRD group showed the least CMT change from baseline to final follow-up (ANOVA P<0.001; *post-hoc*: DRT vs. CME P<0.001, DRT vs. SRD P<0.001). Binary logistic regression identified baseline CMT (β=0.012, 95%CI 1.000–1.024, P = 0.043), inner HRF (β=–0.712, 95%CI 0.238–1.011, P = 0.047), and outer HRF (β=–0.797, 95%CI 0.375–1.083, P = 0.031) as independent predictors of visual prognosis.

**Conclusion:**

Anti-VEGF therapy can reduce the number of HRF in the three subtypes of DME. Baseline HRF, especially within the inner and outer retinal layers, are useful prognostic markers for visual acuity. Subtype-specific treatment and monitoring approaches may enhance long-term visual prognosis in DME patients.

## 1 Introduction

Diabetic retinopathy (DR), a major complication of diabetes, ranks as the fifth leading cause of moderate-to-severe visual impairment and blindness among working-age populations globally (1). Diabetic macular edema (DME), a common complication of DR, manifests as fluid accumulation in the macula, leading to retinal thickening and vision loss (2). Optical coherence tomography (OCT), a non-invasive retinal imaging modality, allows for precise qualitative and quantitative assessment of DME-related microstructural changes, facilitating diagnosis and therapeutic monitoring (3). Based on OCT findings, DME is classified into three subtypes: cystoid macular edema (CME), diffuse retinal thickening (DRT), and serous retinal detachment (SRD) (4).

Advancements in OCT technology have identified multiple biomarkers predictive of treatment outcomes (5). While DME typically presents as hyporeflective cystoid spaces, scattered hyperreflective foci (HRF) in retinal layers are observed in some cases (6). HRF are increasingly recognized as biomarkers of edema severity, with higher counts indicating poorer prognosis (7–9). Other prognostic indicators include central macular thickness (CMT), disorganization of retinal inner layers (DRIL), IS/OS disruption, and hard exudates (10, 11).

Intravitreal anti-VEGF therapy remains the first-line treatment of DME. Previous studies have shown that certain biomarkers in OCT can predict the efficacy of anti-VEGF treatment (12, 13). Many previous studies have focused on analyzing the changes in OCT biomarkers before and after anti - VEGF treatment in DME patients. However, whether there are differences among subtypes of DME remains to be further investigated. This study retrospectively analyzed the clinical data of a group of DME patients, compared the therapeutic effects of three types of DME on anti VEGF therapy. We investigated the underlying causes of differences in OCT biomarkers across the three groups and their association with final visual acuity, aiming to provide new insights for personalized diagnosis and treatment of different DME subtypes.

## 2 Materials and methods

### 2.1 Study population

Medical records of DME patients who received anti-VEGF treatment in the Ophthalmology Department of Shandong Provincial Hospital Affiliated to Shandong First Medical University from January 2021 to March 2023 were retrospectively analyzed. The research protocol was reviewed and approved by the Medical Ethics Committee of Shandong Provincial Hospital Affiliated to Shandong First Medical University. Clinical research was strictly conducted in accordance with the Declaration of Helsinki. All patients and their families were informed of the research objectives, design, and precautions before the start of the study and signed informed consent forms.

Inclusion criteria: (1) Patients with type 2 diabetes mellitus aged over 18 years. (2) Patients with newly - diagnosed DME who had never received intravitreal drug injection or laser therapy before. (3) The central foveal retinal thickness was > 250μm. (4) In cases of bilateral eye involvement, if the causes were the same, the eye with the earliest diagnosis was included; if both eyes were affected simultaneously, the right eye of the patient was included. (5) Patients without severe cardiovascular, cerebrovascular, or other systemic diseases and who could cooperate with the surgery. (6) Patients should have received anti VEGF treatment at least 3 times and have been followed up for more than 12 months.

Exclusion criteria: (1) Patients with unclear OCT images due to refractive media opacities. (2) Patients with other concurrent eye diseases, such as glaucoma, uveitis, retinal vein occlusion, age - related macular degeneration, vitreomacular traction, and pathological myopia, or those with a history of vitrectomy. (3) Patients with uncontrolled severe systemic diseases (e.g., uncontrolled hypertension, recent myocardial infarction or cerebrovascular accident, end-stage renal failure) that would preclude the safe administration of intravitreal injections or could significantly compromise the patient’s ability to complete the 12-month follow-up.

### 2.2 Examination methods

All patients underwent a comprehensive ophthalmic examination before injection, including best corrected visual acuity (BCVA), intraocular pressure (IOP), slit-lamp microscopy, color fundus photography, fluorescein fundus angiography, and OCT (Optovue, RTVue-XR Avanti, USA). Among them, BCVA was converted to the logarithm of the minimum angle of resolution (LogMAR) visual acuity. For extremely low visual acuities, such as counting fingers and hand motion, according to previous literature reports, they were assigned values of 1.9 and 2.3, respectively (14).

Based on the results of OCT imaging and color fundus photography, we included central macular thickness (CMT), the presence or absence of disrupted retinal inner layers (DRIL), the integrity of the inner segment/outer segment (IS/OS) layer, the number of hyperreflective foci (HRF), and the presence or absence of hard exudates in the observation indicators. Among them, DRIL was defined as the inability to distinguish the boundary between the ganglion cell layer-inner plexiform layer or the inner plexiform layer-outer plexiform layer within the horizontal range of the retina (15).The disruption of the integrity of the IS/OS layer was manifested as the discontinuity of the hyperreflective band above the retinal pigment epithelium (RPE) layer. HRF was defined as a circular or oval hyperreflective spot with a reflectivity equal to or higher than the RPE band and a diameter of 20-40 μm. When counting, the number of HRF in each retinal layer within the 1 mm area of the fovea was calculated respectively, including the points in the inner retinal layer (from the inner limiting membrane to the inner nuclear layer), the outer retinal layer (from the outer plexiform layer to the ellipsoid zone), and the subretinal space (from the outer segment of the photoreceptor to the RPE). The results of the OCT examination and fundus examination were qualitatively and quantitatively evaluated by two experienced ophthalmologists specializing in fundus diseases.

### 2.3 Grouping

According to the edema type of DME, the patients were divided into three groups: the CME group, the DRT group, and the SRD group. Among them, CME was manifested as intraretinal cystic cavities with low reflectivity, separated by high-reflectivity septa; DRT was manifested as spongy swelling in the macular area of the retina; SRD was manifested as a shallow detachment between the neurosensory retina and the retinal pigment epithelium, forming a clear hyporeflective area. Among them, if the OCT manifestation was DRT accompanied by CME or SRD, it was classified into the CME or SRD group. If DRT, CME, and SRD appeared simultaneously, it was classified into the SRD group (16).

### 2.4 Intravitreal anti - VEGF drug injection

Before the injection, the treated eye was instilled with proparacaine hydrochloride eye drops three times for surface anesthesia. The patient was placed in the supine position. The eye area was routinely disinfected and draped. An adhesive drape was applied to the treated eye, and an eyelid speculum was used to open the eyelid. The conjunctival sac was irrigated with diluted iodine - based antiseptic solution (Type III Anerdian). A 0.05 - ml aliquot of anti-VEGF drug was drawn, and a 30G needle was inserted into the vitreous cavity 4 mm posterior to the temporal limbus of the treated eye marked by a goniometer. The drug was then injected. The injection site was pressed with a wet cotton swab, and the needle was withdrawn, followed by continued pressure for 1 minute. The intraocular pressure (IOP) of the treated eye was estimated by finger palpation (Tn), and the light perception was positive. The pupillary light reflex of the treated eye was examined and found to be sensitive. Ofloxacin eye ointment was applied to the treated eye, and the eye was bandaged.

During the follow-up period, according to the “3 + PRN” protocol, patients received intravitreal aflibercept injections once a month for 3 consecutive months starting from the initiation of treatment. Subsequently, they had regular monthly follow-up visits and were treated as needed. Throughout the course of therapy, all included patients consistently received intravitreal aflibercept injections and did not switch to other anti-VEGF medications. During the follow-up, patients underwent BCVA, IOP, slit-lamp examination, color fundus photography, and OCT examination for analysis and comparison. If necessary, the panretinal photocoagulation (PRP) could be considered. All patients were followed up for at least 12 months. The baseline conditions of the treated eye before the first injection and the changes in BCVA, IOP, CMT, and the number of HRF in each retinal layer at 1 month, 3 months, 6 months, and 12 months after the injection were recorded. The BCVA of the patients at the last follow - up during the post-injection observation period was compared with the pre-injection BCVA. An improvement or decline of ≥ 1 line in visual acuity was regarded as visual improvement or decline, and no change compared with the pre-injection state was considered visual stability.

### 2.5 Statistical analysis

The statistical analysis was conducted using SPSS 25.0 statistical software. Categorical data were presented as the number of cases (n) and percentages (%), while continuous data were presented as the mean ± standard deviation. One - way analysis of variance (ANOVA) was used to determine whether there were statistically significant differences in the distribution of continuous data among clinical characteristics and ocular parameters of different types of DME. After performing a significant overall one-way ANOVA, *post-hoc* pairwise comparisons were conducted using the Least Significant Difference (LSD) test (or other appropriate tests such as Bonferroni, where applicable) to identify which specific group differences contributed to the overall significance. The chi - square test was employed to analyze whether there were statistically significant differences in the distribution of categorical data. Binary logistic regression analysis was used to examine the factors influencing the visual outcome of DME patients after anti - VEGF treatment. A P - value less than 0.05 was considered to indicate a statistically significant difference.

## 3 Results

### 3.1 Baseline characteristics

**Table 1** shows that Baseline structural biomarkers distinguished the DRT group from CME and SRD: DRIL was present in only 6.82% of DRT eyes, compared to 72.22% of CME and 66.67% of SRD eyes (ANOVA P<0.001; *post-hoc*: DRT vs. CME P<0.001, DRT vs. SRD P<0.001, CME vs. SRD P = 0.563). Similarly, IS/OS disruption was rare in DRT (9.09%) but common in CME (72.22%) and SRD (72.73%) (ANOVA P<0.001; *post-hoc*: DRT vs. CME/SRD P ≤ 0.05). All other baseline factors were balanced across groups (all P>0.05), ensuring no confounding by these variables.

**Table 1:** Baseline data of patients with different types of DME.

	DRT (n=44)	CME (n=36)	SRD (n=33)	P-value (ANOVA)	*Post-hoc* P-value		
					DRTvsCME	DRTvsSRD	CMEvsSRD
Sex(case)							
male	20	17	20	0.377			
female	24	19	13				
Eye(case)							
right	27	18	14	0.245			
left	17	18	19				
Age(years)	59.23 ± 8.75	57.44 ± 9.45	53.36 ± 9.08	0.150			
Duration of diabetes(years)	13.66 ± 7.80	12.25 ± 7.28	13.12 ± 4.76	0.602			
Hypertension(%)	20(45.45)	11(30.56)	13(39.39)	0.396			
IOP(mmHg)	15.43 ± 3.77	16.47 ± 4.20	17.06 ± 3.80	0.058			
DRIL(%)	3(6.82)	26(72.22)	22(66.67)	<0.001	<0.001	<0.001	0.563
IS/OS disruption(%)	4(9.09)	26(72.22)	24(72.73)	<0.001	<0.001	<0.001	0.050
Hard exudates(%)	33(75.00)	29(80.56)	27(81.82)	0.731			
PRP during the treatment period(%)	5(11.36%)	7(19.4%)	5(15.2%)	0.604			

IOP, intraocular pressure; DRIL, disorganization of the retinal inner layers; IS/OS, inner segment/outer segment; PRP, panretinal photocoagulation.

### 3.2 Comparison of BCVA (logMAR) among the three types of DME patients

**Table 2** shows that during the follow-up process, the CME group consistently demonstrated the worst visual acuity (LogMAR) compared to the other two groups (CME vs. DRT/SRD, *post-hoc*: all P<0.05), while the differences between the DRT group and the SRD group were not statistically significant at all time points (DRT vs. SRD, *post-hoc*: all P>0.05).

**Table 2:** Comparison of BCVA (LogMAR) among the 3 groups.

	DRT(n=44)	CME(n=36)	SRD(n=33)	P-value (ANOVA)	*Post-hoc* P-value		
					DRTvsCME	DRTvsSRD	CMEvsSRD
baseline	0.48 ± 0.48	0.72 ± 0.45	0.44 ± 0.22	0.004	0.011	0.660	0.005
1 month	0.42 ± 0.42	0.59 ± 0.33	0.36 ± 0.22	0.003	0.031	0.420	0.006
3 months	0.29 ± 0.30	0.52 ± 0.33	0.31 ± 0.25	<0.001	0.001	0.797	0.003
6 months	0.21 ± 0.14	0.55 ± 0.36	0.26 ± 0.21	<0.001	<0.001	0.477	<0.001
12 months	0.20 ± 0.22	0.43 ± 0.36	0.26 ± 0.25	0.005	0.001	0.353	0.017

BCVA, best corrected visual acuity.

**Figure 1:**
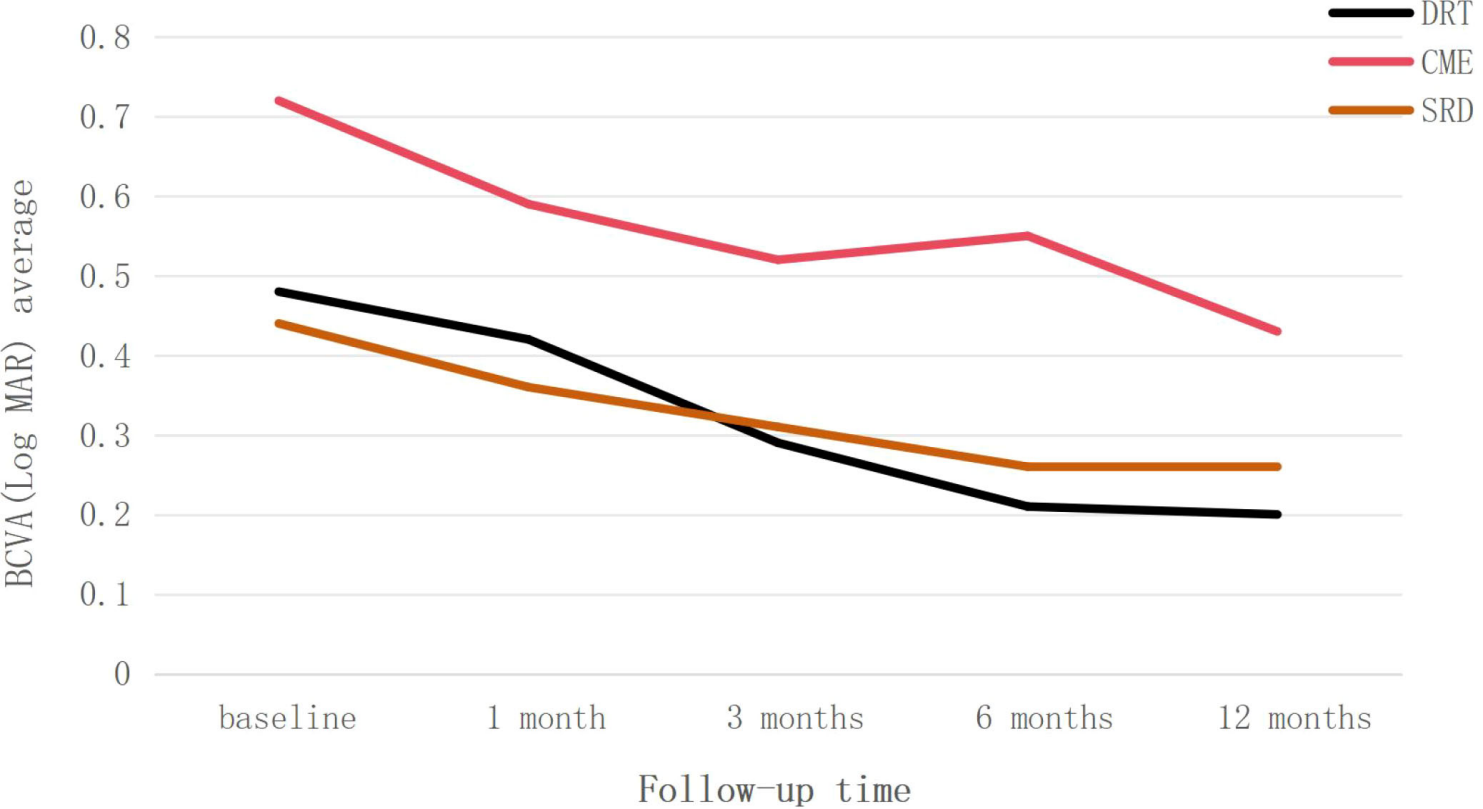
Trend of best-corrected visual acuity (BCVA) changes, presented as LogMAR, over the 12-month follow-up period for the three diabetic macular edema subtypes: diffuse retinal thickening (DRT), cystoid macular edema (CME), and serous retinal detachment (SRD). Data are presented as average values.

**Figure 1** demonstrates that during the follow-up period, the DRT and SRD groups generally showed a trend of visual improvement. CME group exhibited visual acuity fluctuations during follow-up. BCVA showed improvement between 1–3 months after injection, declined from 3–6 months, and improved again from 6–12 months after injection. However, at all follow-up time points, the mean BCVA in the CME group remained worse than that of the other two groups.

**Table 3** outlines that in the DRT group (n=44), 79.55% of patients showed improved vision, 13.64% remained stable, and 6.81% experienced deterioration; in the CME group (n=36), 86.11% showed improvement, 11.11% remained stable, and 2.78% experienced deterioration; in the SRD group (n=33), 72.73% showed improvement, 6.06% remained stable, and 21.21% experienced deterioration. A statistically significant difference was observed in the distribution of visual changes among the three groups (p = 0.031). The proportion of vision deterioration in the SRD group was significantly higher than that in the DRT and CME groups (*post-hoc*: DRT vs. SRD p = 0.033; CME vs. SRD p = 0.009).

**Table 3:** The proportion of the patients with the BCVA increased, stable and decreased.

	Cases	Change in visual acuity (%)			P-value	*Post-hoc* P-value		
		Increased	Stable	Decreased		DRTvsCME	DRTvsSRD	CMEvsSRD
DRT	44	35(79.55)	6(13.64)	3(6.81)	0.031	1.000	0.033	0.009
CME	36	31(86.11)	4(11.11)	1(2.78)				
SRD	33	24(72.73)	2(6.06)	7(21.21)				

### 3.3 Changes in CMT among the three types of DME patients

**Table 4** shows that at baseline, the DRT group exhibited the lowest CMT values compared to the other two groups (ANOVA P<0.001; *post-hoc*: DRT vs. CME P<0.001, DRT vs. SRD P<0.001), while no statistically significant difference was observed between CME and SRD (*post-hoc*: CME vs. SRD P = 0.172). At the 6-month follow-up, significant differences emerged, primarily driven by the comparison between DRT and CME (ANOVA P = 0.017; *post-hoc*: p=0.006). The overall change in CMT from baseline to the final follow-up differed significantly among the groups, with the SRD group showing the least change (ANOVA P<0.001; *post-hoc*: DRT vs. CME P<0.001, DRT vs. SRD P<0.001). At other time points during the observation period, there were no significant differences in the CMT levels among the three groups of patients (P > 0.05).

**Table 4:** Changes in CMT among the 3 groups.

	DRT(n=44)	CME(n=36)	SRD(n=33)	P-value (ANOVA)	*Post-hoc* P-value		
					DRTvsCME	DRTvsSRD	CMEvsSRD
baseline	291.95 ± 41.09	442.36 ± 165.32	483.70 ± 145.79	<0.001	<0.001	<0.001	0.172
post-injection							
1 month	284.93 ± 43.20	346.75 ± 131.90	338.55 ± 123.10	0.140			
3 months	302.48 ± 93.72	330.03 ± 94.94	363.21 ± 134.12	0.073			
6 months	294.57 ± 70.73	351.78 ± 110.93	333.45 ± 92.05	0.017	0.006	0.067	0.407
12 months	282.95 ± 49.35	305.92 ± 62.32	289.82 ± 66.01	0.148			
changes	-9.00 ± 49.17	-136.44 ± 172.51	-193.88 ± 153.75	<0.001	<0.001	<0.001	0.076

CMT, central macular thickness.

### 3.4 Changes in the number of HRF among the three types of DME patients

**Table 5** shows that from baseline to the final follow-up, the DRT group consistently demonstrated the lowest inner HRF values (ANOVA, all P<0.05; *post-hoc*: DRT vs. CME, all P<0.05; DRT vs. SRD, all P<0.05), while no statistically significant differences were observed between the CME and SRD groups (*post-hoc*: CME vs. SRD, all P>0.05). The change in inner HRF was smallest in the DRT group (ANOVA P<0.001; *post-hoc*: DRT vs. CME P = 0.001, DRT vs. SRD P = 0.005).

**Table 5:** Changes in the number of HRF among 3 groups.

	DRT(n=44)	CME(n=36)	SRD(n=33)	P-value (ANOVA)	*Post-hoc* P-value		
					DRTvsCME	DRTvsSRD	CMEvsSRD
HRF:inner							
baseline	2.98 ± 1.45	5.58 ± 1.87	5.70 ± 2.42	<0.001	<0.001	<0.001	0.805
post-injection							
1 month	2.57 ± 1.45	3.86 ± 1.78	4.24 ± 1.64	<0.001	0.001	<0.001	0.330
3 months	2.52 ± 1.76	3.47 ± 1.59	4.27 ± 2.04	<0.001	0.020	<0.001	0.067
6 months	2.27 ± 1.56	3.50 ± 1.92	3.70 ± 1.78	0.001	0.002	0.001	0.640
12 months	1.91 ± 1.22	3.06 ± 1.69	3.33 ± 1.76	<0.001	0.001	<0.001	0.457
changes	-1.07 ± 1.44	-2.53 ± 2.14	-2.36 ± 2.34	<0.001	0.001	0.005	0.730
HRF:outer							
baseline	1.59 ± 1.63	3.31 ± 1.58	2.97 ± 2.21	<0.001	<0.001	0.001	0.442
post-injection							
1 month	1.23 ± 1.48	2.06 ± 1.51	3.64 ± 2.67	<0.001	0.056	<0.001	0.001
3 months	1.07 ± 1.21	1.75 ± 1.46	3.15 ± 2.56	<0.001	0.09	<0.001	0.001
6 months	1.06 ± 1.12	1.94 ± 1.35	2.39 ± 2.30	0.004	0.018	0.001	0.252
12 months	0.66 ± 0.99	1.47 ± 0.97	1.97 ± 1.81	<0.001	0.006	<0.001	0.109
changes	-0.93 ± 1.45	-1.83 ± 1.34	-1.00 ± 2.81	0.011	0.039	0.878	0.075
HRF: SRF					P-Value (vs. baseline)		
baseline	-	-	1.36 ± 1.43	-	-		
post-injection							
1 month	-	-	1.03 ± 1.65	-	0.369		
3 months	-	-	0.36 ± 0.99	-	<0.001		
6 months	-	-	0.00 ± 0.00	-	<0.001		
12 months	-	-	0.09 ± 0.29	-	<0.001		

HRF, hyperreflective foci; SRF, subretinal fluid.

For outer HRF, significant baseline differences were also observed, with the DRT group showing the lowest counts (ANOVA P<0.001; *post-hoc*: DRT vs. CME P<0.001, DRT vs. SRD P = 0.001). After injection, the SRD group consistently exhibited higher outer HRF counts than the other two groups at 1 and 3 months (*post-hoc*: DRT vs. SRD, all P<0.001; CME vs. SRD, all P = 0.01). The DRT group showed lower outer HRF counts than the other two groups at 6 and 12 months (ANOVA, all P<0.05; *post-hoc*: DRT vs. CME, all P<0.05; DRT vs. SRD, all P<0.05).

For HRF in SRF, data were only available for the SRD group. A significant decrease from baseline was observed at 3, 6, and 12 months (all P<0.001), with near-complete resolution by 6 months (**Table 5**).

### 3.5 Analysis of the effect of influence on vision prognosis

Binary logistic regression was employed to analyze risk factors influencing the improvement in visual acuity following anti-VEGF therapy in DME patients. The independent variables included the DRIL, IS/OS disruption, baseline CMT, inner HRF, and outer HRF, with visual acuity improvement serving as the dependent variable. The dependent variable was visual acuity improvement at 12 months.

The coding was as follows: DRIL (no = 0, yes = 1), IS/OS disruption (no = 0, yes = 1), and visual acuity improvement (defined as a gain of ≥ 1 line in LogMAR BCVA; no = 0, yes = 1). The results demonstrated that baseline CMT, inner HRF, and outer HRF were independent predictive factors for visual prognosis in DME patients following anti-VEGF treatment (**Table 6**).

**Table 6:** Factors associated with visual acuity improvement in DME patients after anti-VEGF treatment.

	β	SE	Wald x2 value	P value	OR value	95%CI
DRIL	-1.11	1.331	0.695	0.404	0.33	0.024-4.477
IS/OS disruption	1.56	1.245	1.569	0.210	4.759	0.414-54.648
Baseline CMT	0.012	0.006	3.561	0.043	1.012	1.000-1.024
HRF:inner	-0.712	0.369	3.721	0.047	0.490	0.238-1.011
HRF:outer	-0.797	0.37	4.641	0.031	0.579	0.375-1.083

DRIL, disorganization of the retinal inner layers; IS/OS, inner segment/outer segment; CMT, central macular thickness; HRF, hyperreflective foci.

## 4 Discussions

**Figure 2:**
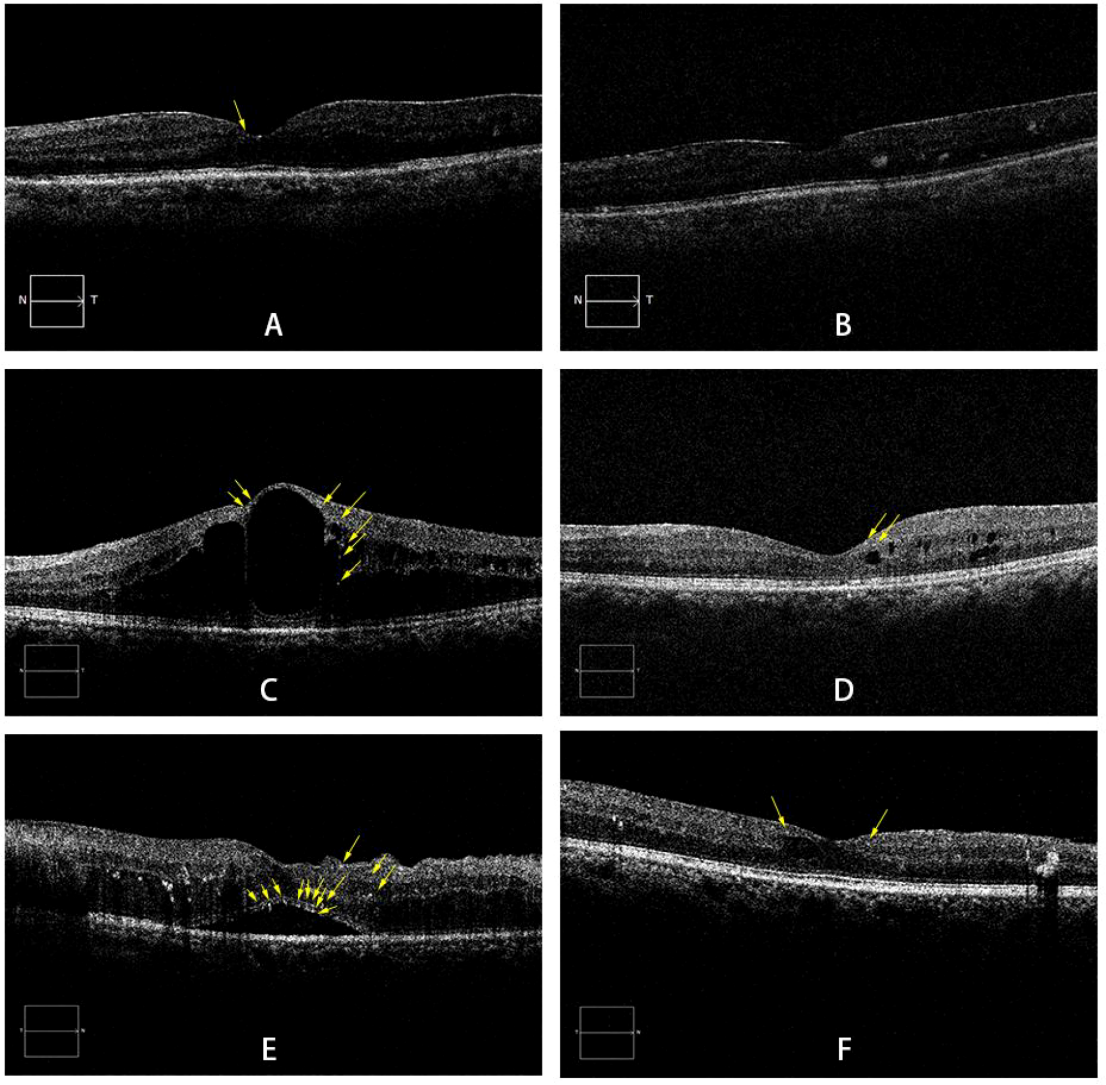
Representative SD-OCT images of DME subtypes. **(A, B)** A 60-year-old male with DRT: pre-injection BCVA of LogMAR 0.52 **(A)**; BCVA of LogMAR 0.30 at the final follow-up **(B)**. **(C, D)** A 52-year-old male with CME: pre-injection BCVA of LogMAR 1.0 **(C)**; BCVA of LogMAR 0.52 at the final follow-up **(D)**. **(E, F)** A 59-year-old female with SRD: pre-injection BCVA of LogMAR 0.3 **(E)**; BCVA of LogMAR 0.10 at the final follow-up **(F)**. Yellow arrows indicate HRF. DME: diabetic macular edema; DRT: diffuse retinal thickening; CME:cystoid macular edema; SRD: serous retinal detachment; HRF: hyperreflective foci.

This study demonstrated that all three subtypes of DME patients exhibited improved BCVA from baseline after anti-VEGF therapy during the 1-year follow-up, confirming the efficacy of the treatment (**Figure 2**). Analysis of visual acuity trends revealed distinct response patterns among the subtypes: The DRT group attained better final visual acuity. Although the CME group showed a greater degree of visual improvement, their visual acuity level was relatively poor at the final follow-up, and a decline in visual acuity was observed at 6 month after treatment. This phenomenon holds certain clinical significance. In addition, the SRD group exhibited the lowest rate of visual improvement and the highest probability of visual deterioration. To identify factors influencing visual outcomes following anti-VEGF therapy in DME patients, we collected baseline and post-injection follow-up observational indicators and analyzed their correlations.

The presence of DRIL indicates disruption and dysfunction of bipolar cells, amacrine cells, or horizontal cells, and may suggest interruption of the visual signal transmission pathway from photoreceptors to ganglion cells, leading to visual impairment (17). Previous studies have identified DRIL as a predictive biomarker for anti-VEGF treatment response in DME patients, with its baseline presence associated with poor visual prognosis (5, 18). As another anatomical biomarker reflecting inflammatory severity in DME, the integrity of the IS/OS layer serves as a crucial indicator of photoreceptor and retinal pigment epithelial health. Maheshwary et al. reported that within the central 500μm of the fovea, every 1% increase in IS/OS disruption corresponded to a reduction of 0.3 ETDRS letters in VA (19). However, our logistic regression analysis did not reveal a significant correlation between baseline DRIL/IS/OS disruption and visual outcomes. We speculate that this discrepancy with previous findings may stem from the rapid resolution of macular edema following anti-VEGF therapy, which likely dominates visual improvement. The potential benefits of structural recovery might be overshadowed by the pronounced positive effects of edema reduction.

In this study, we analyzed the characteristics of HRF in different retinal layers and conducted a multivariate analysis in combination with the final visual acuity. We observed that the DRT group consistently demonstrated fewer HRF (e.g., inner retinal layer: 1.91 ± 1.22 at 12 months vs. 3.06 ± 1.69 in CME, P = 0.001; 3.33 ± 1.76 in SRD, P<0.001) in both retinal layers than the other two groups at all follow-up time points. Pre-injection data showed that the CME and SRD groups had a higher number of HRF in both retinal layers than the DRT group (e.g., inner retinal layer: 2.98 ± 1.45 in DRT vs. 5.58 ± 1.87 in CME P<0.001, DRT vs. 5.70 ± 2.42 in SRD P<0.001). According to the binary logistic regression, we found that the lower baseline number HRF could predict the favorable visual outcomes after treatment (inner retinal HRF:β=-0.712, 95%CI 0.238–1.011, P = 0.047; outer retinal HRF:β=-0.797, 95%CI 0.375–1.083, P = 0.031). This is consistent with the results of many previous studies (16, 20). Some studies have shown that HRF may represent the extravasation of lipoproteins secondary to inflammation or activated microglia, and serve as surrogate markers of retinal inflammation (21, 22). Compared to CME and SRD, DRT is primarily characterized by diffuse thickening between retinal layers with relatively uniform fluid distribution and relatively intact retinal structure and morphology. Therefore, the number of HRF in DRT tends to be lower than that in the other two subtypes, and the visual prognosis is relatively better. Relevant studies indicate that CME exhibit more pronounced microaneurysms compared to those with DRT or SRD, attributable to VEGF induction. The presence of microaneurysms facilitates direct extravasation of blood constituents into cystoid spaces, promoting retinal thickening and cystic alterations (23). As for the SRD group, fluid and exudates flow into the subretinal space through discontinuities in the outer retina, leading to the accumulation of SRF. This process may attract macrophage activation from capillaries and endogenous microglia, resulting in an increased number of HRF spanning the full retinal thickness. The greater inflammatory factor load and more severe retinal structural damage ultimately contribute to poor visual prognosis (24, 7). At baseline compared to the final follow-up during the observation period, we observed the significant reduction in HRF across retinal layers in eyes with CME and SRD, suggesting a pronounced anti-inflammatory effect of anti-VEGF therapy in this subtype. However, this substantial reduction in HRF did not make final visual acuity better, likely due to the more pronounced microaneurysm-mediated vascular leakage, the concurrent high prevalence of irreversible structural disruptions like DRIL and IS/OS disruption, which ultimately limited visual recovery. This does not negate the predictive value of HRF for visual outcomes, rather, it indicates that its predictive value is not absolute. HRF must be evaluated in conjunction with other anatomical structures—such as DRIL and IS/OS disruption—as these structural factors determine the potential for functional recovery of the retina.

Binary logistic regression analysis showed that a high baseline CMT (β=0.012, 95%CI 1.000–1.024, P = 0.043) was a factor predicting visual acuity improvement in DME patients after anti-VEGF injection. Arf S et al. (25) believed that DRT often occurs in the early stage of DME. Due to the relatively complete morphology and structure of the retina, the prognosis is often good. In the study by Kang SW et al. (26), they believed that compared with CME and SRD, patients with CME had lower visual acuity, thicker CMT, and retinal detachment at the fovea of the macula often led to cystoid changes. It was speculated that SRD often occurred before CME in DME. Therefore, DRT, SRD, and CME may represent different processes of DME. The later the disease course, the worse the prognosis is often. When combining the final visual acuity of the three types of DME with the CMT, the CMT of the DRT group before the anti-VEGF treatment and 6 months after the treatment was smaller than that of the other two groups (e.g., 294.57 ± 70.73 μm at 6 months vs. 351.78 ± 110.93 μm in CME, P = 0.012; 333.45 ± 92.05 μm in SRD, P = 0.079), and the decrease in CMT was also the smallest, but the final visual acuity was better (0.20 ± 0.22 LogMAR at 12 months vs. 0.43 ± 0.36 in CME, P = 0.001; 0.26 ± 0.25 in SRD, P = 0.353). In contrast, the CME group showed the great reduction in CMT but ended up with the poorest BCVA. This dissociation between anatomical and functional outcomes suggests that, within the context of different DME subtypes, the integrity of the retinal microstructure may play a more decisive role in determining final visual acuity than the reduction of retinal thickness alone. Based on our data, we propose that patients in the DRT group were able to achieve excellent functional recovery even with minimal anatomical improvement. We hypothesize that visual function depends not only on the resolution of edema—as measured by CMT—but more critically on the preservation of photoreceptor integrity and the capacity of the inner retinal layers to transmit visual signals—a capacity that remained largely intact in the DRT group but was compromised in the CME group. The CME group exhibited severe structural disorganization at baseline—such as DRIL and IS/OS disruption (**Table 1**). Consequently, despite substantial fluid resolution, persistent impairment in the visual pathway led to the worst final BCVA. Therefore, as a late form of DME, relying solely on anti-VEGF drug treatment for CME may not achieve the desired results. Felinski EA believed that suppressing inflammatory factors with glucocorticoids to reduce the swelling of Müller cells and reduce their liquefaction and necrosis may be more effective than anti-VEGF treatment (27).

In addition, we found that at the 6th month after receiving anti-VEGF treatment, recurrence occurred in the CME group, manifested as a decrease in visual acuity (e.g., 0.55 ± 0.36 LogMAR at 6 months vs. 0.52 ± 0.36 LogMAR at 3 months), accompanied by an increase in CMT (351.78 ± 110.93 μm at 6 months vs. 330.03 ± 94.94 μm at 3 month) and the number of HRF in each retinal layer. As the first-line treatment for DME, although anti-VEGF drugs have significant effects, up to 50% of patients still have persistent or recurrent macular edema after multiple intravitreal anti-VEGF drug injections, and their visual acuity does not improve or even decreases (28). In a study involving 51 patients with the CME type and a total of 54 eyes, the observation eyes receiving anti-VEGF treatment were observed for up to 2 years. Whether the cystoid spaces at the fovea were accompanied by hyperreflective walls or not, a decrease in visual acuity and an increase in CMT occurred around the 6th month after the initial intravitreal anti-VEGF treatment (29). Hsia NY et al. (30) retrospectively analyzed DME patients who received dexamethasone intravitreal implant (DEX) treatment after at least 3 months of ineffective intravitreal anti-VEGF treatment. They found that in the first month after switching to DEX treatment, the patients’ BCVA and CMT were significantly improved compared with the baseline. Combined with previous research results, visual acuity and OCT inflammatory markers (e.g., HRF) at 6 months post-injection should be closely monitored: subtype-specific changes (e.g., CMT/HRF rebound in CME) represent preliminary signals of suboptimal sustained response to anti-VEGF therapy, which may warrant consideration of medication transition—consistent with prior evidence of improved outcomes with switching in similar patient groups.

The limitation of this study lies in the relatively short follow-up period, which may cause certain deviations in the research results, and the relatively small sample size, resulting in insufficient persuasiveness of the research results. Future prospective studies comparing continued anti-VEGF vs. medication transition (e.g., to steroids) in patients with 6-month rebound/persistent HRF are needed to validate this clinical recommendation.

## 5 Conclusion

Anti-VEGF therapy can reduce the number of HRF in the three subtypes of DME. Baseline HRF, especially within the inner and outer retinal layers, are useful prognostic markers for visual acuity. Subtype-specific treatment and monitoring approaches may enhance long-term visual prognosis in DME patients.

## Data Availability

The original contributions presented in the study are included in the article/supplementary material. Further inquiries can be directed to the corresponding author.
